# Colorectal Cancer Incidence and Mortality Disparities in New Mexico

**DOI:** 10.1155/2014/239619

**Published:** 2014-01-02

**Authors:** Richard M. Hoffman, David K. Espey, Robert L. Rhyne, Melissa Gonzales, Ashwani Rajput, Shiraz I. Mishra, S. Noell Stone, Charles L. Wiggins

**Affiliations:** ^1^Department of Medicine, University of New Mexico School of Medicine, Albuquerque, NM 87131, USA; ^2^Medicine Service, New Mexico VA Health Care System, 1501 San Pedro Drive SE, Albuquerque, NM 87108, USA; ^3^University of New Mexico Cancer Center, Albuquerque, NM 87106, USA; ^4^Department of Family and Community Medicine, University of New Mexico School of Medicine, Albuquerque, NM 87131, USA; ^5^Division of Cancer Prevention and Control, Centers for Disease Control and Prevention, Atlanta, GA 30341, USA; ^6^Department of Surgery, University of New Mexico School of Medicine, Albuquerque, NM 87131, USA; ^7^Department of Pediatrics, University of New Mexico School of Medicine, Albuquerque, NM 87131, USA

## Abstract

*Background.* Previous analyses indicated that New Mexican Hispanics and American Indians (AI) did not experience the declining colorectal cancer (CRC) incidence and mortality rates observed among non-Hispanic whites (NHW). We evaluated more recent data to determine whether racial/ethnic differences persisted. *Methods.* We used New Mexico Surveillance Epidemiology and End Results data from 1995 to 2009 to calculate age-specific incidence rates and age-adjusted incidence rates overall and by tumor stage. We calculated mortality rates using National Center for Health Statistics' data. We used joinpoint regression to determine annual percentage change (APC) in age-adjusted incidence rates. Analyses were stratified by race/ethnicity and gender. *Results.* Incidence rates continued declining in NHW (APC −1.45% men, −1.06% women), while nonsignificantly increasing for AI (1.67% men, 1.26% women) and Hispanic women (0.24%). The APC initially increased in Hispanic men through 2001 (3.33%, *P* = 0.06), before declining (−3.10%, *P* = 0.003). Incidence rates declined in NHW and Hispanics aged 75 and older. Incidence rates for distant-stage cancer remained stable for all groups. Mortality rates declined significantly in NHW and Hispanics. *Conclusions.* Racial/ethnic disparities in CRC persist in New Mexico. Incidence differences could be related to risk factors or access to screening; mortality differences could be due to patterns of care for screening or treatment.

## 1. Introduction

Colorectal cancer incidence and mortality rates have steadily declined in the United States (USA) over the past three decades [1]. The 2013 Annual Report to the Nation on the Status of Cancer estimated that from 1998 to 2009 the average annual percent change in colorectal cancer incidence declined by 2.6% in men and 2.1% in women [2]. Mortality rates also declined for both men and women by an average annual percent change of 2.9%. Declining incidence rates were observed for all racial and ethnic groups. However, the incidence and mortality declines were less for Hispanics than non-Hispanics [2].

Interpreting the national data on Hispanics is complicated because the race and ethnicity categories are not mutually exclusive and there are many distinct Hispanic subgroups. However, data from the population-based New Mexico Tumor Registry (NMTR) and a Surveillance, Epidemiology, and End Results (SEER) registry are sufficiently detailed to provide mutually exclusive rates for the major population groups in the state—non-Hispanic whites, Hispanics, and American Indians [3]. Investigators analyzing New Mexico colorectal cancer data previously observed marked disparities that countered national trends. Chao and colleagues found that the New Mexico colorectal cancer incidence and mortality rates in non-Hispanic whites declined from 1969 to 1994 in parallel with national trends but increased among Hispanics and American Indians, particularly among men [4]. We have updated the analyses of NMTR data through 2009 to determine whether there were persistent racial/ethnic differences in colorectal cancer incidence rates, tumor characteristics (stage, anatomic subsites), and mortality rates.

## 2. Materials and Methods

We used data from the NMTR to calculate incidence rates from 1995 to 2009 for non-Hispanic whites, Hispanics, and American Indians. All cases were New Mexico residents diagnosed with invasive cancer of the colon (International Classification of Diseases for Oncology—Third Edition (ICD-O-3) topography codes C18.0, C18.2-C18.9, and C26.0), rectosigmoid junction (ICD-O-3 topography codes C19.9), or rectum (ICD-O-3 site codes C20.9). Cases with lymphoma (ICD-O-3 histology codes 9590-9989), mesothelioma (ICDO-3 histology code 9050–9055), and Kaposi's sarcoma (ICDO-3 histology code 9140) were excluded from the analysis. Cancers of the appendix (ICD-O-3 topography code C18.1) were also excluded so that results would be more comparable with previously reported New Mexico data [4].

The NMTR primarily determined race/ethnicity from abstracting medical records. Hispanic ethnicity was assigned on the basis of specific statements in medical records augmented by identifying Spanish surnames and maiden names (when available) [3]. American Indian ancestry was documented from medical records and through routine linkages with administrative records from the Indian Health Service [5]. Cases with American Indian ancestry were allocated to the American Indian category regardless of Hispanic ethnicity. Estimating cancer death and incidence rates for American Indians can be problematic because race is often misclassified in vital statistics and cancer registries [6, 7]. The most accurate death and incidence rates are based on the Indian Health Service (IHS) Contract Health Services Delivery Area (CHSDA) counties, which generally contain federally recognized tribal lands or are adjacent to tribal lands [5]. In New Mexico, 97.4% of the New Mexican AI/AN population resides in CHSDA counties (Melissa Jim, MPH, IHS, personal correspondence).

The NMTR registry staff documented primary cancer site from pathology reports and other medical records, coding sites according to the prevailing ICD-O edition at the time of diagnosis (ICD-O-2 from 1995–2000, ICD-O-3 from 2001–2009). All ICD-O-2 cases were converted to ICD-O-3 to ensure comparability over time. To examine colorectal cancers by anatomic subsites, we classified cancers of the ascending colon, hepatic flexure, transverse colon, and splenic flexure (ICD-O-3 codes C18.0, C18.2-C18.5) as being right-sided; cancers of the descending and sigmoid colon (ICD-O-3 C18.6-18.7) as left-sided; and cancers of the rectosigmoid junction (ICD-O-3 C19.9) and rectum (ICD-O-3 C20.0) as rectosigmoid. Stage of disease at diagnosis for colorectal cancer was described using the categories of localized (confined to the colon or rectum), regional (extending to nodes or pericolonic tissue), and distant (metastatic) [8].

Cause of death was based on death certificate data for New Mexico residents compiled by the National Center for Health Statistics [9]. Cause of death was coded according to the Ninth Revision (1995–1998) and Tenth Revision (1999–2009) of the International Classification of Diseases (ICD) [10]. We analyzed colorectal cancer deaths coded as 153, 154.0-154.1, or 159.0 in the Ninth Revision or coded as C18, C19, C20, or C26.0 in the Tenth Revision.

### 2.1. Data Analysis

We used the National Cancer Institute's SEER*Stat software system [11] to calculate age-specific colorectal cancer incidence rates and to calculate average annual age-adjusted incidence and mortality rates (by the direct method) [12] using the US 2000 standard population [13]. All rates were expressed per 100,000 population with 95% confidence intervals (CIs) calculated by SEER*Stat methods [14]. We also calculated age-adjusted incidence rates for cancer stage at diagnosis and for specific anatomic subsites within the colon and rectum. We stratified rates by gender and race/ethnicity (non-Hispanic white, Hispanic, and American Indian) for the time periods 1995–1999, 2000–2004, and 2005–2009.

We assessed temporal changes in annual age-adjusted incidence rates with joinpoint regression techniques [15] using statistical software developed by the National Cancer Institute [16]. We derived denominators for rate calculations from annual US Census Bureau population estimates stratified by race, sex, and 5-year age group [17].

We calculated rate ratios and 95% confidence intervals to compare rates across racial/ethnic groups and over time [18]. We used chi-square analyses to compare the proportions of cases diagnosed at various stages and tumor subsites across gender and racial/ethnic groups [12]. For all analyses, we considered *P* values < 0.05 to be statistically significant.

## 3. Results

During the time period from 1995 through 2009, a total of 11,017 colorectal cancer cases meeting our eligibility criteria were diagnosed in the New Mexico non-Hispanic white (*n* = 6995), Hispanic (*n* = 3523), and American Indian (*n* = 499) populations, including 5950 cases in men and 5067 cases in women. Age-adjusted incidence rates were significantly higher among men than women for all time periods (*P* < 0.001).

**Figure 1:**
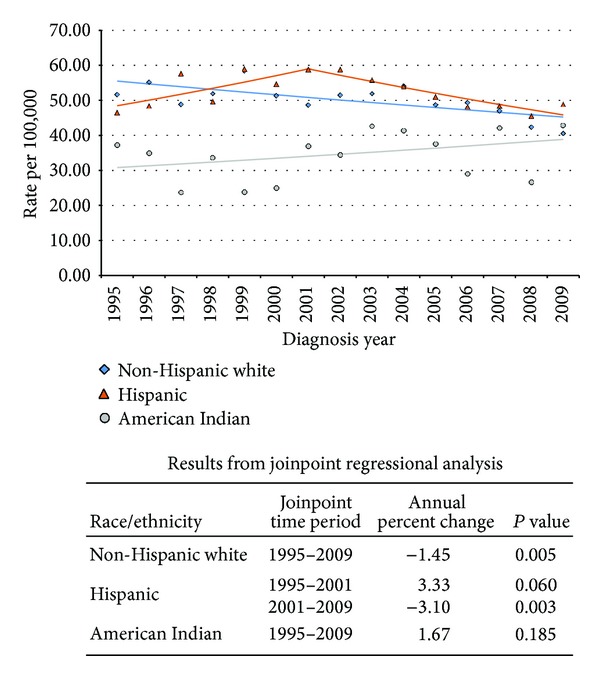
New Mexico colorectal cancer male incidence joinpoint by race/ethnicity, 1995–2009.

**Figure 2:**
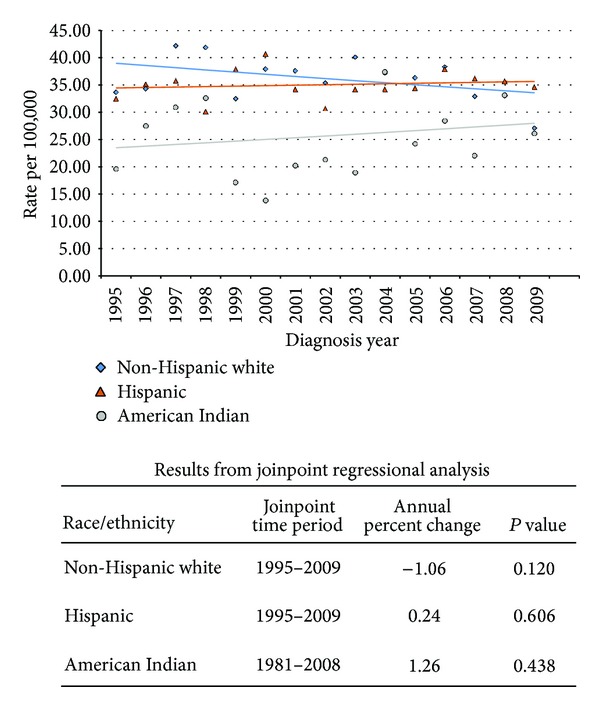
New Mexico colorectal cancer female incidence joinpoint by race/ethnicity, 1995–2009.

New Mexico experienced marked racial/ethnic differences in age-adjusted annual colorectal cancer incidence rates based on joinpoint regression analyses (Figures 1 and 2). In 1995, cancer incidence rates were highest for both men and women among non-Hispanic whites and lowest among American Indians. Subsequently, cancer incidence rates declined among both non-Hispanic white men (annual percent change (APC) −1.45%, *P* = 0.005) and women (APC −1.06%, *P* = 0.12).

In contrast, cancer incidence rates modestly increased among American Indian men (APC 1.67%, *P* = 0.19) and women (APC 1.26%, *P* = 0.43), though neither change was statistically significant. Incidence rates also nonsignificantly increased among Hispanic women (APC 0.24%, *P* = 0.61), eventually exceeding those of non-Hispanic white women by the end of 2009. Joinpoint regression analyses identified two distinct trends in cancer incidence rates among Hispanic men. From 1995 to 2001, the incidence rate nonsignificantly increased (APC 3.33%, *P* = 0.06), peaking at 59.2 per 100,000 in 2001. Subsequently, the incidence rate significantly declined (APC −3.10%, *P* = 0.003), though from 1998 to 2009 Hispanic men had the highest colorectal cancer incidence rate of any New Mexico population.

Average age-specific incidence rates by time period, race/ethnicity, and gender are shown in Table 1. In all racial/ethnic groups, age-specific incidence rates substantially increased with advancing age. Age-specific colorectal cancer incidence rates increased over time among Hispanic men and non-Hispanic whites younger than 50, though absolute increases were only 1.5 to 2.5/100,000. Incidence rates for those aged 50 to 64 remained constant over time but declined among non-Hispanic whites aged 65 to 74 and among non-Hispanic whites and Hispanic men aged 75 and older.

**Figure 3:**
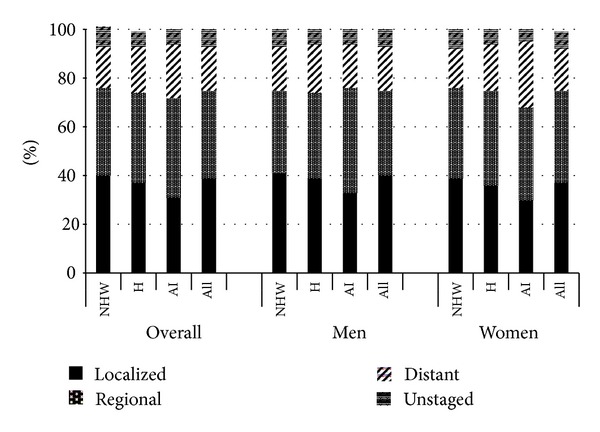
New Mexico colorectal cancer stage distribution by race/ethnicity and gender, 1995–2009. NHW: non-Hispanic white, H: Hispanic, and AI: American Indian. Columns do not all sum to 100% due to rounding.

Stage-specific incidence rates varied little over time by racial/ethnic group for either men or women (Table 2). During the time period from 1995 to 2009, the proportion of cancers diagnosed at localized stage was significantly higher for non-Hispanic whites (40%) compared to either Hispanics (37%, *P* = 0.01) or American Indians (31%, *P* < 0.001) (Figure 3). Overall, men were more likely than women to be diagnosed at localized stage, 40% versus 37%, *P* = 0.007. Within racial/ethnic groups, a significant gender difference was observed only among non-Hispanic whites: men were more likely than women to be diagnosed at localized stage, 41% versus 39%, *P* = 0.05. Meanwhile, American Indians were more likely to be diagnosed at distant stage (22%) than non-Hispanic whites (17%, *P* = 0.003), though not Hispanics (19%, *P* = 0.59). Among American Indians, women were more likely than men to be diagnosed at distant stage, 27% versus 18%, *P* = 0.03.

**Figure 4:**
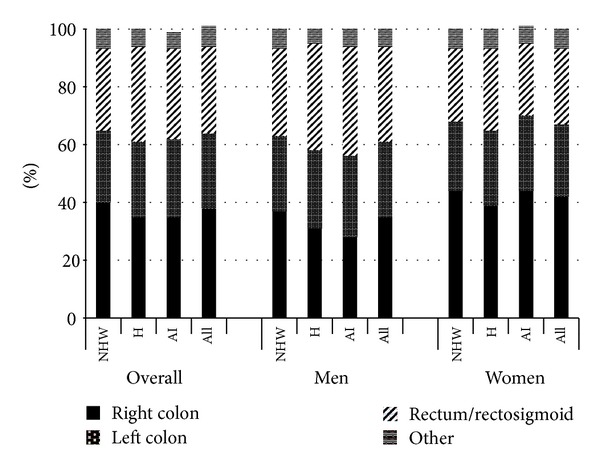
New Mexico colorectal cancer anatomic subsite distribution by race/ethnicity and gender, 1995–2009. NHW: non-Hispanic white, H: Hispanic, and AI: American Indian. Columns do not all sum to 100% due to rounding.

Incidence rates by anatomic subsites also varied little over time by racial/ethnic group for either men or women (Table 3). Incidence rates for right-sided tumors, which were lower among American Indians and Hispanics, declined only among Hispanic men. Incidence rates for left-sided cancers, which were similar across all groups, declined only among non-Hispanic whites. The overall proportion of right-sided cancers diagnosed during the entire study period was significantly higher among non-Hispanic whites (40%) compared to either American Indians (35%, *P* = 0.05) or Hispanics (35%, *P* < 0.0001) (Figure 4). The overall proportion of right-sided cancers was significantly higher among women than men, 42% versus 35%, *P* < 0.0001. Within racial/ethnic groups, significant differences in the proportions of right-sided cancers between women and men were observed for non-Hispanic whites (44% versus 39%, *P* < 0.0001) and Hispanics (39% versus 31%, *P* < 0.0001).

During the time period from 1995 through 2009, a total of 4088 colorectal cancer deaths occurred in the New Mexico non-Hispanic white (*n* = 2525), Hispanic (*n* = 1371), and American Indian (*n* = 192) populations, including 2175 in men and 1913 in women. Average age-adjusted mortality rates (Table 4) were significantly higher among men than women for all time periods (*P* < 0.001). Combined mortality rates have consistently been higher among Hispanics (and lower among American Indians) compared to non-Hispanic whites, though differences were significant only during the time period from 2000 to 2004. During that time period, mortality rates in men were significantly higher among Hispanics and lower among American Indians, while American Indians had the lowest mortality rate among women. Combined mortality rates declined among non-Hispanic whites and Hispanics but not for American Indians. However, few deaths occurred among American Indians and estimated rates were unstable. Mortality rates declined significantly among Hispanic men.

## 4. Discussion

Colorectal cancer incidence and mortality trends in New Mexico since 1995 show marked disparities by race and ethnicity. While age-adjusted incidence and mortality rates have steadily decreased among non-Hispanic whites similar to national data, trends have been quite different for Hispanics and American Indians. A previous report on colorectal cancer in New Mexico from 1969 to 1994 showed declining incidence and mortality rates among non-Hispanic whites, but increasing incidence and mortality among minority populations, particularly for men [4]. The most striking finding in the recent time period was the persistently increasing cancer incidence rates among Hispanic men from 1995 to 2001, followed by a significantly declining rate. Nonetheless, Hispanic men have had the highest colorectal cancer incidence rate of any New Mexican population since 1998. Meanwhile, incidence rates have remained stable among Hispanic women and American Indians. Colorectal cancer mortality rates have been decreasing among non-Hispanic whites and Hispanics, though not among American Indians.

National data have not shown the age-adjusted colorectal cancer incidence and mortality disparities between non-Hispanic whites and Hispanics that we observed in New Mexico [2]. Hispanics are a very heterogeneous population, and results from New Mexico, where Hispanics trace their ancestry back to Mexico or Spain, might not necessarily be comparable to other Hispanic subgroups included in national data. Risk factors and health behaviors could explain some of the disparities in incidence and mortality. Hispanics in New Mexico have a higher prevalence of risk factors for colorectal cancer than non-Hispanic whites, including obesity, diabetes, physical inactivity, and tobacco use [19]. Behavioral Risk Factor Surveillance Survey (BRFSS) data from New Mexico also indicate that Hispanics are less likely than non-Hispanic whites to be current with colorectal cancer screening [20], which could contribute to differences in both incidence and mortality. However, mortality rates among non-Hispanic whites began declining in the 1980s, before cancer screening was routinely recommended or widespread [4]. Barriers to accessing care, which could result in more advanced stage at diagnosis and/or receipt of less intensive treatment, might also account for some of the disparities. We previously reported that racial/ethnic differences in screening uptake could be largely explained by adjusting for socioeconomic factors [19].

Chao and colleagues previously showed a significant 3.6% average percent increase in colorectal cancer incidence rates among Hispanic men from 1969 through 1994 [4]. We observed a 3.3% annual percent change in colorectal cancer incidence in Hispanic men from 1995 through 2001. Although this change was not significant, it was comparable to the increase observed during the previous time period. However, from 2001 to 2009, the incidence rate then declined significantly among Hispanic men. These temporal patterns among Hispanic men are puzzling. Higher uptake of screening years earlier could have prevented cancers by detecting and removing precancerous polyps. Conversely, decreased screening uptake during the most recent time period could have led to fewer cancers being detected—though rates would eventually increase in the future with a shift towards more advanced-stage cancers. Historically, Hispanic men have lower screening rates than non-Hispanic whites according to BRFSS, but rates are highest among Hispanic men over 70 [19]. Men in this age range are eligible for Medicare screening and the decline in cancer incidence was most notable among older Hispanic men suggesting a possible screening effect.

A marked decrease in risk factors for colorectal cancer occurring many years in the past could also account for the recent declining incidence. However, BRFSS data suggest that Hispanic men have equal or higher prevalence of obesity, diabetes, and smoking than Hispanic women and non-Hispanic whites [19].

Overall, American Indians had the lowest age-adjusted incidence and mortality rates in New Mexico. Rates increased slightly during the study period, but the changes were not significant. However, sample sizes were small and point estimates imprecise. National data have shown declining incidence and mortality rates, though with substantial regional variation [7]. Screening prevalence among New Mexican American Indians is low; [20] obesity, diabetes, and physical inactivity are common among Southwestern American Indians though the prevalence of smoking is low [21].

Colorectal cancer incidence rates were highest in the oldest age group but generally declined over time, most notably among non-Hispanic whites and Hispanics. This could be due to increased uptake of screening in the previous decade [22]. Medicare began funding screening fecal blood testing in 1998 and then colonoscopy in 2001 [23].

The age-adjusted incidence rates by stage were fairly similar across racial/ethnic groups and constant over time. However, non-Hispanic whites had the highest proportions of cancers diagnosed at localized stage while American Indians had the highest proportion diagnosed at distant-stage. These differences are most likely due to screening uptake, though could also be related to behavioral, socioeconomic, genetic, and environmental factors.

In contrast to the 1969 to 1994 New Mexico data [4], we did not observe any changes in the incidence rates of right-sided colon cancers (which previously increased among Hispanics and American Indian women). Furthermore, the incidence rates of left-sided cancers were no longer increasing among Hispanic and American Indian men. However, only non-Hispanic whites had significant declines in the incidence rates of left-sided cancers, which are the most readily prevented by screening [24].

Our analyses had some potential limitations. The NMTR has developed effective strategies for determining Hispanic ethnicity, but misclassification is possible, particularly for married women with Hispanic surnames. National data suggest that an apparent American Indian over white mortality advantage is reversed by adjusting for misclassification in the American Indian population [25]. However, almost all New Mexican American Indians reside in CHSDA counties where IHS data provide the most accurate racial classifications. Linkages between the National Death Index and the Indian Health Services databases have shown that AI/AN misclassification rate for New Mexico was less than 4% during the study period (Melissa Jim, MPH, IHS, personal correspondence). The validity of cause of death coding on death certificates, particularly for American Indians, has been problematic in the past. However, during the study period estimates for specific causes of death were considered to have become more accurate because fewer deaths among American Indians were coded as “symptoms and ill-defined conditions” [26].

## 5. Conclusions

In updating an earlier report on colorectal incidence and mortality in New Mexico we continue to observe disparities in incidence and mortality rates. Minority populations were not experiencing the same rate declines observed in non-Hispanic whites, particularly for incidence rates. Further research is needed to address these disparities, including whether differences in cancer incidence can be attributed to differences in screening uptake or prevalence of risk factors. Either explanation could lead to targeted prevention efforts. Mortality differences could be due to unequal receipt of guideline-concordant treatment and surveillance protocols. Such findings could reflect barriers to accessing health care, particularly in rural, underserved areas, that need to be addressed in allocating health care resources.

## Figures and Tables

**Table 1:** Average age-specific colorectal cancer incidence rates by time period, race/ethnicity, and gender; New Mexico, 1995–2009.

Age	Time period	Incidence rate (95% CI) by race/ethnicity			Rate ratio (95% CI)	
		Non-Hispanic white	Hispanic	American Indian	H versus NHW	AI versus NHW
Men						
≤50	1995–1999	4.6 (3.5–5.8)	2.8 (2.0–3.8)	4.5 (2.6–7.3)	0.60 (0.40–0.92)	0.99 (0.54–1.74)
	2000–2004	5.5 (4.3–6.9)	4.5 (3.5–5.7)^a^	4.6 (2.7–7.3)	0.82 (0.58–1.16)	0.84 (0.47–1.42)
	2005–2009	7.1 (5.7–8.8)^a^	4.3 (3.4–5.5)^a^	8.0 (5.5–11.3)	0.62 (0.44–0.85)	1.13 (0.73–1.71)
50–64	1995–1999	84.3 (74.9–94.4)	83.8 (71.2–98.1)	70.7 (45.8–104.4)	1.00 (0.81–1.21)	0.84 (0.53–1.26)
	2000–2004	83.0 (74.5–92.2)	96.7 (84.4–110.3)	85.8 (61.6–116.4)	1.16 (0.98–1.38)	1.03 (0.73–1.43)
	2005–2009	81.4 (73.6–89.9)	86.0 (75.5–97.6)	69.8 (50.1–94.7)	1.06 (0.90–1.24)	0.86 (0.61–1.18)
65–74	1995–1999	235.5 (213.1–259.5)	249.3 (215.0–287.5)	100.6 (52.0–175.7)	1.06 (0.89–1.26)	0.43 (0.22–0.75)
	2000–2004	241.0 (218.5–265.2)	255.6 (223.0–291.6)	107.0 (61.2–173.7)	1.06 (0.89–1.25)	0.44 (0.25–0.73)
	2005–2009	187.9 (168.7–208.7)^a^	247.1 (216.9–280.5)	131.2 (83.1–196.8)	1.31 (1.11–1.55)	0.70 (0.44–1.06)
75+	1995–1999	364.4 (330.6–400.7)	352.3 (298.8–412.5)	131.8 (63.2–242.4)	0.97 (0.80–1.17)	0.36 (0.17–0.67)
	2000–2004	322.4 (292.9–354.2)	343.9 (297.1–396.0)	181.5 (105.7–290.5)	1.07 (0.90–1.27)	0.56 (0.32–0.91)
	2005–2009	285.8 (259.6–313.9)^a^	227.0 (193.5–264.6)^a^	116.3 (61.9–198.9)	0.79 (0.66–0.95)	0.41 (0.22–0.70)

Women						
≤50	1995–1999	4.6 (3.5–5.8)	3.5 (2.6–4.6)	3.8 (2.1–6.4)	0.77 (0.52–1.13)	0.84 (0.44–1.52)
	2000–2004	5.5 (4.3–6.9)	4.4 (3.4–5.6)	4.5 (2.7–7.1)	0.79 (0.56–1.13)	0.82 (0.46–1.39)
	2005–2009	7.1 (5.6–8.7)^a^	4.5 (3.5–5.6)	5.7 (3.6–8.5)	0.63 (0.45–0.88)	0.80 (0.48–1.29)
50–64	1995–1999	56.7 (49.2–64.9)	57.7 (47.7–69.2)	39.1 (22.8–62.5)	1.02 (0.80–1.28)	0.69 (0.39–1.13)
	2000–2004	56.3 (49.5–63.7)	50.7 (42.2–60.4)	40.9 (25.9–61.4)	0.90 (0.72–1.12)	0.73 (0.45–1.12)
	2005–2009	56.2 (50.0–63.1)	62.6 (53.9–72.3)	43.7 (29.5–62.3)	1.11 (0.92–1.34)	0.78 (0.51–1.13)
65–74	1995–1999	160.3 (142.9–179.2)	128.9 (106.3–154.8)	104.9 (60.0–170.4)	0.80 (0.64–1.00)	0.65 (0.37–1.08)
	2000–2004	151.3 (134.3–169.9)	134.0 (112.3–158.6)	91.4 (54.2–144.5)	0.89 (0.72–1.09)	0.60 (0.35–0.97)
	2005–2009	124.5 (109.7–140.7)^a^	147.3 (125.7–171.6)	94.2 (59.0–142.6)	1.18 (0.97–1.45)	0.76 (0.47–1.17)
75+	1995–1999	254.4 (231.5–278.9)	229.3 (193.9–269.4)	152.6 (88.9–244.3)	0.90 (0.74–1.09)	0.60 (0.35–0.97)
	2000–2004	261.3 (239.3–284.6)	245.1 (211.9–282.1)	107.2 (60.0–176.8)	0.94 (0.79–1.11)	0.41 (0.23–0.68)
	2005–2009	221.3 (201.9–242.1)^a^	198.6 (171.9–228.3)	127.6 (80.0–193.3)	0.90 (0.76–1.06)	0.58 (0.36–0.88)

^a^ *P* < 0.05 comparing time period versus 1995–1999.

**Table 2:** Average age-adjusted colorectal cancer incidence rates by stage, time period, gender, and race/ethnicity; New Mexico, 1995–2009.

Stage	Time period	Incidence rate (95% CI) by race/ethnicity			Rate ratios (95% CI)	
		Non-Hispanic white	Hispanic	American Indian	H versus NHW	AI versus NHW
Men						
Localized	1995–1999	21.2 (19.3–23.2)	20.5 (17.7–23.6)	9.7 (5.6–15.3)	0.97 (0.82–1.15)	0.46 (0.26–0.73)
	2000–2004	20.5 (18.8–22.4)	20.9 (18.4–23.6)	9.2 (5.6–13.9)	1.02 (0.87–1.28)	0.45 (0.27–0.69)
	2005–2009	19.4 (17.8–21.2)	19.0 (16.8–21.3)	14.0 (10.0–19.0)	0.98 (0.84–1.13)	0.72 (0.51–0.99)
Regional	1995–1999	18.1 (16.4–20.0)	19.4 (16.7–22.3)	14.6 (9.8–20.9)	1.07 (0.89–1.27)	0.81 (0.53–1.17)
	2000–2004	18.7 (17.0–20.5)	21.0 (18.4–23.8)	16.1 (11.4–22.1)	1.12 (0.96–1.32)	0.86 (0.60–1.20)
	2005–2009	14.8 (13.3–16.3)^a^	15.1 (13.2–17.2)^a^	11.8 (8.3–16.3)	1.02 (0.86–1.21)	0.80 (0.55–1.12)
Distant	1995–1999	9.6 (8.3–11.0)	10.1 (8.2–12.3)	4.9 (2.4–8.9)	1.06 (0.82–1.34)	0.51 (0.24–0.94)
	2000–2004	8.3 (7.2–9.5)	10.1 (8.4–12.1)	7.5 (4.4–11.8)	1.22 (0.97–1.54)	0.91 (0.52–1.46)
	2005–2009	8.5 (7.4–9.7)	9.6 (8.1–11.3)	5.1 (2.8–8.3)	1.12 (0.91–1.39)	0.59 (0.33–0.99)

Women						
Localized	1995–1999	14.6 (13.2–16.1)	11.5 (9.6–13.5)	4.3 (2.2–7.6)	0.78 (0.64–0.95)	0.30 (0.15–0.52)
	2000–2004	13.7 (12.4–15.1)	11.9 (10.2–13.8)	7.9 (5.0–11.8)	0.87 (0.72–1.04)	0.58 (0.36–0.87)
	2005–2009	13.7 (12.5–15.2)	13.4 (11.8–15.3)	9.2 (6.3–12.8)^a^	0.98 (0.83–1.15)	0.67 (0.45–0.94)
Regional	1995–1999	13.9 (12.5–15.4)	13.2 (11.3–15.4)	13.0 (8.8–18.2)	0.95 (0.79–1.14)	0.93 (0.62–1.33)
	2000–2004	14.5 (13.2–16.0)	14.3 (12.4–16.4)	9.7 (6.5–13.7)	0.99 (0.83–1.16)	0.67 (0.44–0.96)
	2005–2009	12.2 (11.0–13.5)	12.9 (11.3–14.7)	6.5 (4.1–9.6)^a^	1.06 (0.90–1.26)	0.53 (0.33–0.80)
Distant	1995–1999	5.9 (5.0–6.9)	6.5 (5.2–8.1)	7.7 (4.5–12.2)	1.10 (0.83–1.44)	1.30 (0.73–2.12)
	2000–2004	6.1 (5.2–7.1)	6.3 (5.1–7.7)	4.6 (2.6–7.5)	1.03 (0.79–1.32)	0.76 (0.42–1.27)
	2005–2009	5.5 (4.7–6.4)	6.8 (5.7–8.2)	7.1 (4.7–10.3)	1.24 (0.97–1.58)	1.29 (0.83–1.93)

^a^ *P* < 0.05 comparing time period versus 1995–1999.

**Table 3:** Average age-adjusted colorectal cancer incidence rates by anatomic site, time period, gender, and race/ethnicity; New Mexico, 1995–2009.

Anatomic site	Time period	Incidence rate (95% CI) by race/ethnicity			Rate ratios (95% CI)	
		Non-Hispanic white	Hispanic	American Indian	H versus NHW	AI versus NHW
Men						
Right side	1995–1999	19.2 (17.4–21.2)	17.5 (14.9–20.4)	10.0 (5.9–15.6)	0.92 (0.75–1.10)	0.52 (0.31–0.82)
	2000–2004	20.4 (18.7–22.3)	17.7 (15.4–20.3)	9.4 (5.9–14.2)	0.87 (0.73–1.02)	0.46 (0.29–0.70)
	2005–2009	17.0 (15.5–18.7)	14.1 (12.3–16.2)^a^	8.7 (5.6–12.7)	0.83 (0.70–0.98)	0.51 (0.33–0.75)
Left side	1995–1999	14.5 (13.0–16.2)	13.9 (11.6–16.4)	10.6 (6.3–16.4)	0.96 (0.78–1.17)	0.73 (0.43–1.15)
	2000–2004	12.8 (11.5–14.3)	15.8 (13.6–18.2)	9.4 (5.9–14.0)	1.23 (1.02–1.48)	0.73 (0.45–1.11)
	2005–2009	10.8 (9.6–12.1)^a^	12.3 (10.6–14.2)	9.4 (6.2–13.6)	1.13 (0.94–1.37)	0.87 (0.56–1.28)
Rectum and rectosigmoid junction	1995–1999	15.7 (14.1–17.4)	18.6 (16.0–21.5)	9.4 (5.8–14.4)	1.19 (0.98–1.42)	0.60 (0.36–0.93)
	2000–2004	15.1 (13.6–16.7)	20.2 (17.7–22.8)	14.5 (9.9–20.3)	1.33 (1.13–1.57)	0.96 (0.65–1.37)
	2005–2009	14.5 (13.1–16.1)	17.8 (15.7–20.0)	11.9 (8.4–16.4)	1.22 (1.04–1.43)	0.82 (0.57–1.14)

Women						
Right side	1995–1999	15.2 (13.8–16.7)	13.0 (11.1–15.2)	10.1 (6.5–14.8)	0.86 (0.71–1.03)	0.66 (0.42–0.99)
	2000–2004	16.6 (15.2–18.1)	13.7 (11.8–15.7)	9.7 (6.5–13.7)	0.82 (0.70–0.97)	0.58 (0.39–0.84)
	2005–2009	14.4 (13.1–15.8)	14.6 (12.9–16.5)	12.7 (9.3–16.9)	1.02 (0.86–1.18)	0.88 (0.63–1.19)
Left side	1995–1999	10.8 (9.6–12.2)	9.2 (7.6–11.0)	6.9 (4.0–11.0)	0.85 (0.68–1.05)	0.64 (0.37–1.03)
	2000–2004	8.6 (7.6–9.8)^a^	9.5 (8.0–11.2)	7.1 (4.4–10.7)	1.11 (0.89–1.36)	0.83 (0.51–1.27)
	2005–2009	7.4 (6.4–8.4)^a^	8.0 (6.7–9.4)	5.1 (3.0–8.0)	1.08 (0.87–1.34)	0.69 (0.40–1.10)
Rectum and rectosigmoid junction	1995–1999	8.7 (7.6–9.9)	9.9 (8.2–11.8)	7.2 (4.1–11.6)	1.14 (0.91–1.42)	0.83 (0.47–1.36)
	2000–2004	9.1 (8.0–10.3)	9.2 (7.8–10.9)	5.5 (3.3–8.7)	1.01 (0.82–1.25)	0.61 (0.35–0.97)
	2005–2009	9.5 (8.4–10.7)	9.6 (8.2–11.1)	5.2 (3.2–7.9)	1.00 (0.82–1.21)	0.54 (0.33–0.84)

^a^ *P* < 0.05 comparing time period versus 1995–1999.

H: Hispanic, NWH: non-Hispanic white, and AI: American Indian.

**Table 4:** Average age-adjusted colorectal cancer mortality rates by gender, time period, and race/ethnicity; New Mexico, 1995 to 2009.

Gender	Time period	Mortality rates (95% CI) per 100,000 by race/ethnicity			Rate ratios (95% CI)	
		Non-Hispanic white	Hispanic	American Indian	H versus NHW	AI versus NHW
Overall	1995–1999	17.4 (16.3–18.6)	19.6 (17.7–21.5)	13.7 (10.3–17.8)	1.12 (0.99–1.26)	0.79 (0.59–1.03)
	2000–2004	16.6 (15.6–17.8)	19.6 (17.9–21.4)	10.2 (7.7–13.3)	1.18 (1.06–1.32)	0.62 (0.46–0.81)
	2005–2009	15.5 (14.5–16.5)^a^	16.6 (15.2–18.1)^a^	14.4 (11.5–17.8)	1.08 (0.96–1.20)	0.93 (0.74–1.16)

Male	1995–1999	20.8 (18.8–22.8)	24.2 (21.0–27.7)	14.9 (9.7–21.6)	1.17 (0.98–1.38)	0.72 (0.46–1.05)
	2000–2004	20.1 (18.3–22.0)	25.1 (22.2–28.2)	13.6 (9.1–19.3)	1.25 (1.07–1.45)	0.68 (0.45–0.97)
	2005–2009	18.4 (16.8–20.1)	20.0 (17.7–22.5)^a^	15.5 (11.0–21.1)	1.09 (0.93–1.26)	0.84 (0.59–1.16)

Female	1995–1999	14.5 (13.2–16.0)	16.0 (13.9–18.5)	12.8 (8.6–18.3)	1.10 (0.93–1.31)	0.88 (0.58–1.28)
	2000–2004	13.8 (12.5–15.2)	15.2 (13.3–17.4)	8.0 (5.1–11.8)	1.11 (0.93–1.30)	0.58 (0.37–0.87)
	2005–2009	13.2 (12.0–14.6)	13.9 (12.2–15.7)	13.4 (9.8–17.8)	1.05 (0.89–1.23)	1.02 (0.73–1.37)

^a^ *P* < 0.05 comparing time period versus 1995–1999.

H: Hispanic, NWH: non-Hispanic white, and AI: American Indian.
